# Objective measurement of cough in pulmonary fibrosis: a cohort study – ImpaCT

**DOI:** 10.1183/23120541.00310-2024

**Published:** 2024-09-30

**Authors:** Jad Kebbe, Simon P. Hart, Robert J. Kaner, Tejaswini Kulkarni, Lutz Wollin, Carl Coeck, Richard Vinisko, Nina Patel, Marlies S. Wijsenbeek

**Affiliations:** 1 Section of Pulmonary, Critical Care and Sleep Medicine, University of Oklahoma Health Sciences Center, Oklahoma City, OK, USA; 2 Hull York Medical School, University of Hull, Hull, UK; 3 Division of Pulmonary and Critical Care Medicine and Department of Genetic Medicine, Weill Cornell Medicine, New York, NY, USA; 4 University of Alabama at Birmingham, Birmingham, AL, USA; 5 Boehringer Ingelheim Pharma GmbH & Co, KG, Biberach an der Riß, Germany; 6 Boehringer Ingelheim SComm, Brussels, Belgium; 7 Boehringer Ingelheim Pharmaceuticals Inc, Ridgefield, CT, USA; 8 Respiratory Medicine, Erasmus Medical Center, Rotterdam, The Netherlands

## Abstract

Among patients with interstitial lung disease (ILD), cough is a prevalent symptom and can persist over time, with chronic cough having a major impact on patients’ quality of life [1–3]. Moreover, it has been hypothesised that cough may enhance fibrotic remodelling *via* cough-induced mechanical stress [1, 4]. However, while persistent cough is commonly reported in idiopathic pulmonary fibrosis (IPF) and is associated with worse prognosis [1, 3, 5], data are limited on the impact of cough in patients with pulmonary fibrosis other than IPF [2, 5]. This study aimed to assess the frequency and distribution of cough during the day and at night in patients with pulmonary fibrosis other than IPF, using objective cough monitoring as well as patient-reported severity and impact of cough.

## Shareable abstract

**Using both objective cough monitoring and patient-reported outcomes measures, this study describes the burden of cough for patients with non-IPF pulmonary fibrosis** https://bit.ly/3wFm0th

*To the Editor:*

Among patients with interstitial lung disease (ILD), cough is a prevalent symptom and can persist over time, with chronic cough having a major impact on patients’ quality of life [1–3]. Moreover, it has been hypothesised that cough may enhance fibrotic remodelling *via* cough-induced mechanical stress [1, 4]. However, while persistent cough is commonly reported in idiopathic pulmonary fibrosis (IPF) and is associated with worse prognosis [1, 3, 5], data are limited on the impact of cough in patients with pulmonary fibrosis other than IPF [2, 5]. This study aimed to assess the frequency and distribution of cough during the day and at night in patients with pulmonary fibrosis other than IPF, using objective cough monitoring as well as patient-reported severity and impact of cough.

ImpaCT was a multicentre, exploratory cohort study of patients aged ≥18 years who were diagnosed with pulmonary fibrosis other than IPF. Patients with IPF, those with cough due to causes other than fibrosis and individuals with sleep disordered breathing were excluded. Eligible patients were identified by participating physicians in the USA and Europe, and enrolled consecutively. The planned sample size was 100 patients. Patients provided written informed consent before study inclusion and could withdraw at any time. Ethical approval was obtained in each country and for each site from local review boards. The trial was registered with OnderzoekMetMensen.nl (identifier number NL79595.099.22).

Patients attended three study visits over 4 weeks (baseline, V1 and V2) (figure 1), and were followed prospectively from the time of enrolment until the end of the study period, loss to follow-up or death, whichever occurred first. Demographic and baseline variables collected from patient medical records and through routine clinical care were assessed at the initial visit. Cough was measured objectively after V1 and V2 for 24 h using the VitaloJAK cough monitor (Vitalograph, Inc.) [6], and subjectively with patient-reported outcome (PRO) measures (V1 and V2).

**FIGURE 1:**
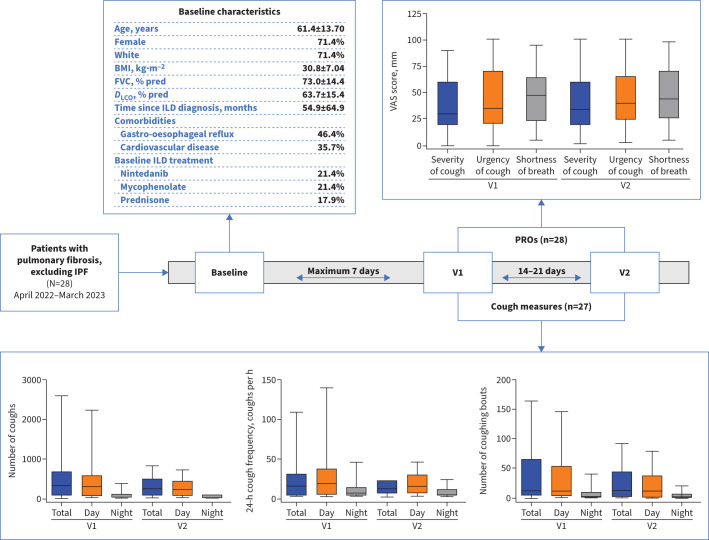
Baseline characteristics, objective cough monitoring and patient-reported outcomes for patients with pulmonary fibrosis. Baseline characteristics are presented as mean±sd or %. Cough measures and patient-reported outcome (PRO) data are shown as median (horizontal line) with interquartile range (box). Whiskers represent range. The cough-evaluable set (n=27) included all patients with at least one recording through the VitaloJAK monitor. The PRO-evaluable set (n=28) included all patients with at least one of the PRO measures completed. In total, 24 patients had data available at both visit (V)1 and V2. BMI: body mass index; *DL*_CO_: diffusing capacity of the lung for carbon monoxide; FVC: forced vital capacity; ILD: interstitial lung disease; IPF: idiopathic pulmonary fibrosis; VAS: visual analogue scale.

The primary objective was to assess frequency and daytime/night-time distribution of cough and coughing bouts (defined as three or more sequential coughs, each cough separated by ≤2 s). Secondary objectives included assessment of PROs, measured by the Leicester Cough Questionnaire (LCQ) and visual analogue scales (VAS) for cough severity, cough urgency and shortness of breath (V1 and V2), Living with Pulmonary Fibrosis (L-PF) cough Impact Item 18 (V1 and V2), and three-item Patient Global Impressions (PGI) scale assessing overall impression of change in cough over the preceding 2 weeks (V2). Log transformation of cough frequency was used for analysis of the relationship between cough frequency and PROs.

Overall, 28 patients (20 female and eight male) were enrolled across the USA and Europe (figure 1). Due to difficulties with enrolment, the study was terminated prematurely. In total, 35.7% of patients had connective tissue disease-associated ILD, 25.0% had fibrotic hypersensitivity pneumonitis, 21.4% had idiopathic nonspecific interstitial pneumonia, 10.7% had unclassifiable ILD and 7.2% had other ILD.

Objective cough counts at V1 and V2 are shown in figure 1. There was intra- and intersubject variation in the distribution of 24-h, daytime and night-time cough frequencies. Of the 24 patients who had recordings at both V1 and V2, the median (interquartile range (IQR)) difference between the two 24-h recording periods was −2.6 (−9.7 to 0.1) coughs per h. The median (IQR) difference in daytime and night-time cough frequency recordings was −4.3 (−11.7 to 0.1) and −0.3 (−3.1 to 1.6), respectively.

VAS scores showed similar trends to cough counts. For the L-PF (Impact Item 18), the proportion of patients with a score ≤2 (negative effect of cough on quality of life) ranged from 46.5% to 64.3% across visits. On the LCQ, total median (IQR) scores were 13.5 (11.8 to 19.3) at V1 and 14.8 (10.3 to 16.8) at V2. In total, 16 (57.1%) patients experienced no impression of change in cough over the preceding 2 weeks using the PGI. For all PRO measures, the total and subdomain scores were similar at V1 and V2.

The overall relationship between cough frequency and VAS items showed moderate correlation, based on Pearson correlation: severity of cough, ρ=0.6 (95% CI 0.3 to 0.7); urgency of cough, ρ=0.5 (95% CI 0.3 to 0.7); shortness of breath, ρ=0.5 (95% CI 0.2 to 0.7).

This study shows a high cough burden observed for patients in this cohort with pulmonary fibrosis other than IPF, as measured objectively by cough frequency over 24 h and subjectively by PROs.

There was a relatively low percentage of patients treated with nintedanib at baseline (21.4%) (figure 1), which may reflect the inclusion criteria (disease progression was not required for enrolment) and/or a lag between nintedanib approval and treatment initiation. The baseline forced vital capacity (FVC) % predicted and diffusing capacity of the lung for carbon monoxide (*D*_LCO_) % predicted suggest that patients were not as severely affected by their disease as those in the INBUILD trial of nintedanib in patients with progressive fibrosing ILD (FVC 69.0% predicted; *D*_LCO_ 46.1% predicted).

The median cough counts in this study (13.1 and 9.7 coughs per h at V1 and V2, respectively) are in line with those observed in IPF studies reporting median baseline objective cough counts of 11.4–16.0 coughs per h [7, 8]. To our knowledge, this is the first study to assess coughing bouts in patients with pulmonary fibrosis other than IPF (figure 1). While the definition of coughing bouts is not standardised, episodes of continuous coughing may impact patient quality of life [9]. Recognising the importance of incorporating patient insights into clinical trial design, we would advocate including a measure of cough bouts as an outcome for future studies [10].

Cough severity VAS scores (mean±sd) at V1 (39.9±26.7) and V2 (41.2±26.5) and 24-h cough frequency (median (IQR)) at V1 (13.1 (2.8–28.4) coughs per h) and V2 (9.7 (4.1–20.8) coughs per h) were similar to what was found for IPF in a systematic literature review [11].

PRO findings were not always consistent with observed patterns of cough measurements for these patients with non-IPF pulmonary fibrosis, similar to observations in patients with IPF [8, 12]. The presence of moderate, but not strong, correlation between cough frequency and VAS scores is consistent with previous reports in patients with chronic cough [13]. This underlines that we likely need to take a multidimensional approach to assessing cough in trials and daily care.

Study limitations include the small sample size, affecting the generalisability of the results. Eligibility criteria were broad and no specific cough threshold was required for inclusion. However, there may have been selection bias, with some investigators potentially enrolling patients with a high degree of cough symptoms.

In conclusion, cough is a significant burden for patients with pulmonary fibrosis other than IPF. To our knowledge, this is the first study to describe cough using objective and subjective metrics in patients with pulmonary fibrosis other than IPF. These data support the inclusion of patients with non-IPF forms of pulmonary fibrosis, in addition to those with IPF, in future cough studies. Such studies may be more attuned to patient experiences to gain insights into cough mechanisms in fibrotic lung diseases and identify effective treatments.

## Data Availability

To ensure independent interpretation of clinical study results and enable authors to fulfil their role and obligations under the International Committee of Medical Journal Editors criteria, Boehringer Ingelheim grants all external authors access to relevant clinical study data. In adherence with the Boehringer Ingelheim Policy on Transparency and Publication of Clinical Study Data, scientific and medical researchers can request access to clinical study data, typically, 1 year after the approval has been granted by major Regulatory Authorities or after termination of the development programme. Researchers should use the https://vivli.org/ link to request access to study data and visit https://www.mystudywindow.com/msw/datasharing for further information.
